# Supplementary material for: Toward a Better Understanding of the Gelation Mechanism of Methylcellulose via Systematic DSC Studies
Source: Polymers (Basel). 2022 Apr 28;14(9):1810. doi: 10.3390/polym14091810 (PMC9105695; doi:10.3390/polym14091810)
Supplement: Supplementary file 1 [file polymers-14-01810-s001.zip › polymers-1703629-supplementary.pdf]

Supplementary data for

Below are presented the dependencies of the parameters of the Asymmetric Double Sigmoid (ADS) functions, which were used for deconvolution of the peaks seen as the thermal effects during heating and cooling. The equation of ADS function is given as:

$$y = A \cdot \frac{1}{1+e^{-\frac{x-x_C+w_1/2}{w_2}}} \cdot \left(1 - \frac{1}{1+e^{-\frac{x-x_C-w_1/2}{w_3}}}\right),$$

where A is amplitude, x_C - center, w_1, w_2, w_3 - widths.

The parameters for the heating and cooling modes are presented in Figure 1 and Figure 2, respectively. In the case of the heating mode, the width parameter w_1 was found as the minor-effect, thus, it was fix as zero in the approximation procedure and the plot is presented.

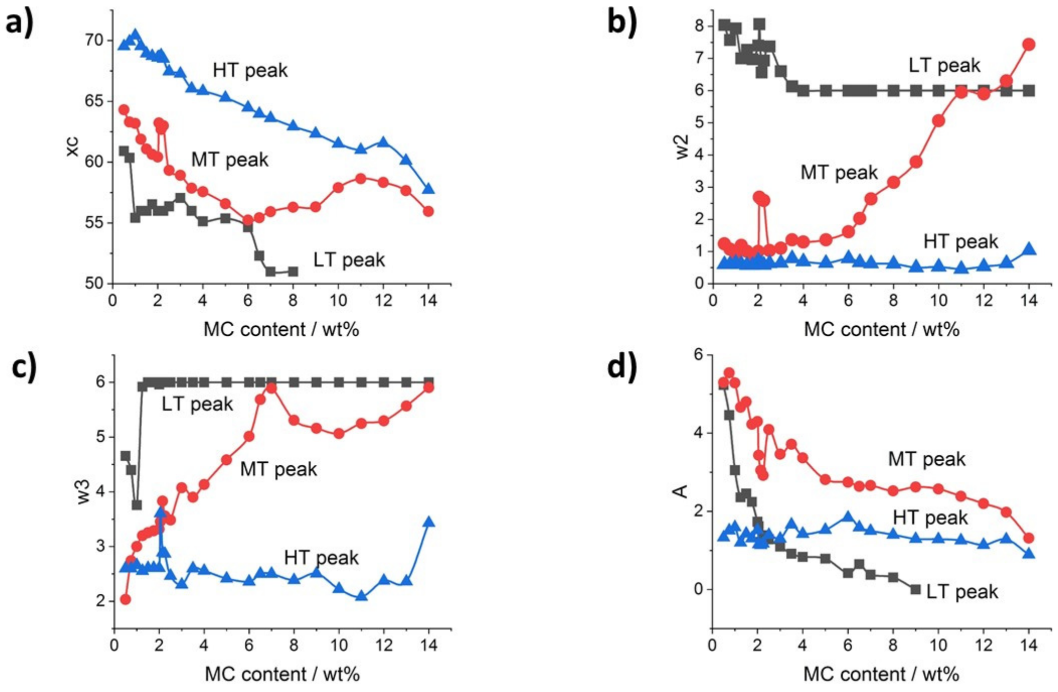

Figure S1. The ADS functions parameters of the peaks determined for the heating mode.

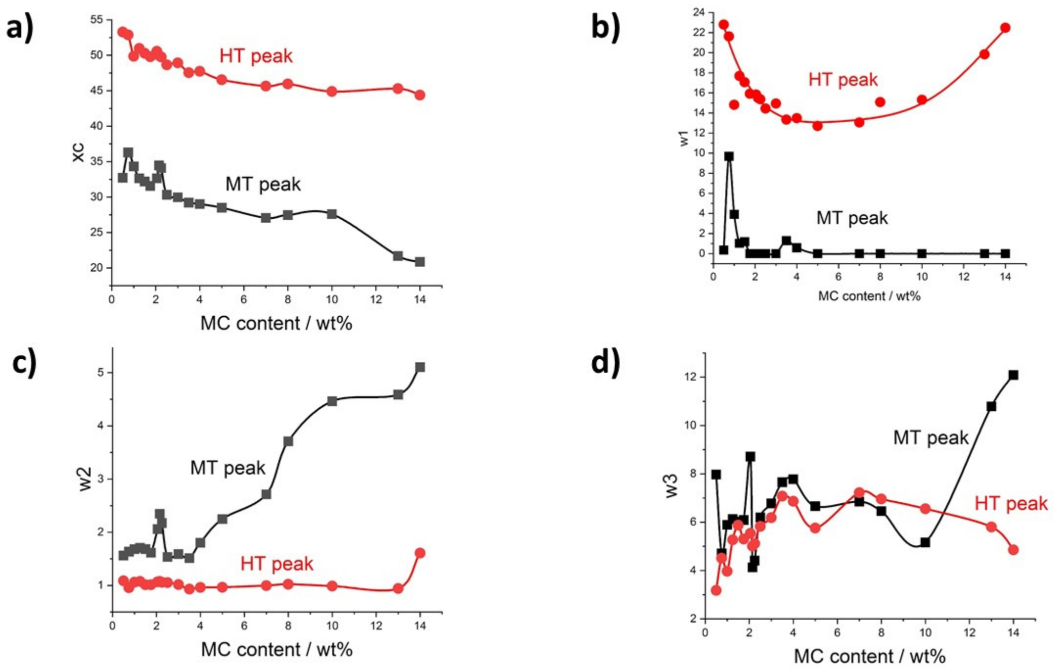

Figure S2. The ADS functions parameters of the peaks determined for the cooling mode.
